# Electronic health records in Romania – window of opportunity in improving population's health?

**DOI:** 10.25122/jml-2022-1032

**Published:** 2022-11

**Authors:** Diana Alecsandra Grad, Dafin Mureșanu

**Affiliations:** 1 RoNeuro Institute for Neurological Research and Diagnostic, Cluj-Napoca, Romania; 2 Department of Public Health, Babes-Bolyai University, Cluj-Napoca, Romania; 3 Department of Neurosciences, Iuliu Hatieganu University of Medicine and Pharmacy, Cluj-Napoca, Romania

In theory, electronic health records (EHRs) contain medical information from all the providers on the services used by patients (screening, preventive, curative, or disease management services). In Romania, since its inception in 2014 (700.000 records) [1] and until late 2021 (the latest available), 16 million EHRs have been created with the help of health professionals and institutions. However, EHRs are poorly used because only a few hospitals are contributing to the data collection process, and most of the data is submitted by general practitioners (GPs) [2]. Promoted as “the ideal instrument to keep track of your family's health”, it contains a summary of medical information for emergencies (*i.e*., transplants, medical devices, allergies, blood, chronic and communicable diseases, current treatment and hospitalizations, and the presence of arteriovenous fistula) and other information on behavioral habits (sleep patterns, alcohol, drug, and tobacco consumption, among others). Furthermore, it allows the patients to see who (and when) accessed their data and to anonymize and choose the data their physician can access [3] (the EHR in Romania is regulated under law 45/2009) [4]. When this project was implemented, there were complaints regarding the lack of cost-effectiveness and feasibility studies of EHRs, unclear judicial framework on logistical infrastructure, access and proprietary rights of the medical data, the distribution of the data collection and data entry process (family doctors being worried that the administrative burden will fall on them), and the alignment of the EHRs with the existing informatics system (SIPE) and the health insurance card (CEAS), among others [1]. The EHRs can be used (if properly implemented and monitored and if the population is educated on how to use them) for multiple purposes (*e.g*., pragmatic trials or risk factors surveillance). The National Health Insurance House is implementing two projects with European funding: eDES (which aims to connect the EHR to all health and health technology providers) and SIGMA SMART (which aims to optimize and improve the data flow of reimbursed medical health services from the National Fund of Social Health Insurance) [5].

Another effort to improve the e-health sector is RegInterMed, which aims to provide an IT platform for a minimum of 100 electronic health registries, to progressively update the data of patients enrolled in those registries and increase the usage of e-health systems, among others [6, 7]. Another project aims to improve the current e-health systems allows the development and implementation of a management system that will provide reports, studies, and analyses aiming to increase the quality and efficiency of services reimbursed by the National Fund of Social Health Insurance [8].

Electronic health records are essential tools in improving health outcomes and delivery processes for patients and providers and represent highly valuable sources of secondary data used in generating observational studies with varying aims and statistical analyses. In addition, if patients are given the option to provide informed consent on being contacted by industry representatives for prospective clinical studies for trial selection and inclusion (often burdensome trial stages money- and timewise), it can accelerate clinical knowledge generation (which will be further employed in assessing the safety, efficacy, effectiveness, and costs) [9–11]. In trials conducted in Nordic countries, investigators named EHR as the most used patient identification source (85%), followed by internal/external referrals [12]. A mixed methods study involving trial recruiters pointed out that most of them are using the EHR as a screening/enrollment tool in combination with the other two methods (future clinic appointments or reaching out to past trial patients) [13]. EHRs are among the most used sources (mobile devices, product and disease registries, and laboratory data) for obtaining real-world evidence (RWE). In order to assist interested parties with this type of evidence, the EMA (European Medicines Agency) and the FDA (U.S. Food and Drug Administration) have issued guidelines on this matter [14–16].

In the context of the COVID-19 pandemic, an abundance of research based on EHRs data has been generated; the first and second years recorded the largest number of citations and publications from EHRs. Thygesen and colleagues have used information in the EHRs (supplemented by additional sources) to map patient pathways, including screening, vaccination, and inpatient and outpatient healthcare elements, and to provide incidence and survival rates [17]. Katsoulis and colleagues estimate the direct and indirect effects of COVID-19 in patients diagnosed with a COVID-19 modifiable risk factor – obesity [18]. Another study by Oetjens and colleagues provides relevant data in establishing kidney disease as a major risk factor for adverse effects associated with a SARS-CoV-2 positive diagnosis [19]. Long COVID has also been the focus of risk and burden of disease factors with a sample composed of self-reported (n=6907) and with a confirmed diagnosis in their EHRs (n=1.1 million) [20]. Dementia – a disease forecasted to affect 152.8 million in 2050 – has been the topic of case-control risk and disparity analysis that used data from 61.9 million EHR, emphasizing the need for tailored COVID-19-related interventions [21, 22]. However, when designing and interpreting EHRs-related research, population representativeness, missing data (as well as quality protocols), threshold interpretation, and availability must be considered [23].

Although EHRs can be used for multiple purposes, EHR-related burnout (due to insufficient training or time conflicting tasks) poses a severe concern in strained healthcare systems [24, 25]. A cross-sectional survey showed that almost half of practicing physicians and a quarter of residents had attributed burnout symptoms to EHR usage [26]. However, another study reports that physician experience and an optimized, available EHRs version account for positive experiences [27]. A systematic review of how EHRs influence physicians' professional identity showed that EHRs are an administrative burden (and has increased the volume of collected data that would benefit the department/ward and not the patient's treatment); a decrease in time also means a decrease of empathetic and respectful interactions. This review also points out that the role of the EHR has shifted from tool to actor in decision-making processes [28]. For example, in Romania, GPs are burdened by the fact that, instead of seeing patients, multiple concurrent applications need to be accessed to complete “a consultation” recognized by the health informatics system [29].

EHRs perceptions among patients are mixed. A survey covering four states showed that patients had positive perceptions of the impact EHRs had on the quality of care [30]. Another study (conducted in the UK) showed that most patients were worried about the security of their health data in the EHRs; however, only 12% opposed the development of national EHRs [31]. A more recent report based on the consultation held by TEHDAS highlighted that people are aware of the benefits of sharing health data but are afraid of how data will be shared with other providers [32].

Romania cannot achieve the full potential of EHRs as long as its health system is deeply fragmented (lack of data from all the providers) and is affected by other weaknesses such as the lack of a period of training dedicated to data collection, management, and analysis, a wide range of software programs used for hospital administrative databases, low number of national registries, and shortage of funding and dedicated personnel [33]. As a result, Romania is behind when it comes to using all the functionalities of EHRs. Nevertheless, there are positive expectations in this regard as one of the objectives of the new strategy is to extend the usage of EHRs to contain information from populational screening programs and the laboratory, ambulatory, rehabilitation, oral health services, as well as home care (and home emergencies and medical devices [34].
